# Promise and challenges of clinical non-invasive T-cell tracking in the era of cancer immunotherapy

**DOI:** 10.1186/s13550-022-00877-z

**Published:** 2022-01-31

**Authors:** Dario Gosmann, Lisa Russelli, Wolfgang A. Weber, Markus Schwaiger, Angela M. Krackhardt, Calogero D’Alessandria

**Affiliations:** 1 Klinik und Poliklinik für Innere Medizin III, Klinikum rechts der Isar, Technische Universität München, Munich, Germany; 2 Klinik und Poliklinik für Nuklearmedizin, Klinikum rechts der Isar, Technische Universität München, Munich, Germany; 3 German Cancer Consortium (DKTK), Partner-Site Munich and German Cancer Research Center (DKFZ), Heidelberg, Germany

**Keywords:** Cancer immunotherapy, Tumor-reactive T-cells, Immuno-imaging, Positron emission tomography, Response evaluation

## Abstract

In the last decades, our understanding of the role of the immune system in cancer has significantly improved and led to the discovery of new immunotherapeutic targets and tools, which boosted the advances in cancer immunotherapy to fight a growing number of malignancies. Approved immunotherapeutic approaches are currently mainly based on immune checkpoint inhibitors, antibody-derived targeted therapies, or cell-based immunotherapies. In essence, these therapies induce or enhance the infiltration and function of tumor-reactive T cells within the tumors, ideally resulting in complete tumor eradication. While the clinical application of immunotherapies has shown great promise, these therapies are often accompanied either by a variety of side effects as well as partial or complete unresponsiveness of a number of patients. Since different stages of disease progression elicit different local and systemic immune responses, the ability to longitudinally interrogate the migration and expansion of immune cells, especially T cells, throughout the whole body might greatly facilitate disease characterization and understanding. Furthermore, it can serve as a tool to guide development as well as selection of appropriate treatment regiments. This review provides an overview about a variety of immune-imaging tools available to characterize and study T-cell responses induced by anti-cancer immunotherapy. Moreover, challenges are discussed that must be taken into account and overcome to use immune-imaging tools as predictive and surrogate markers to enhance assessment and successful application of immunotherapies.

## Introduction

Immunotherapeutic approaches such as immune checkpoint modulation and chimeric antigen receptor (CAR)-T-cell therapy have shown great potential for the treatment of various tumor entities [1]. These therapies often have a variable effect on the immune system resulting in complex patterns of treatment responses as well as toxicity, which are often difficult to predict, diagnose and monitor. Currently, comprehensive predictive as well as surrogate biomarkers for optimal guidance of those therapeutic approaches are missing. Biopsies can often only provide a snapshot information but lack a more general view on pathophysiological or pharmacodynamical aspects. In contrast, imaging methodologies are often more suitable to capture complex three dimensional conditions although reliable and clinically implemented imaging strategies to monitor the variety of immunotherapeutic options and subsequent course of the disease are still not established. This lack of insight may not only misguide therapeutic choices, but can also lead to incorrect assessment of treatment responses including tumor pseudoprogression, resulting in premature termination of the treatment and initiation of alternative, possibly less effective and more harmful alternatives [2]. Therefore, there is an urgent need to develop novel tools to predict, monitor and evaluate the immune response during immunotherapy.

Apart from finding novel surrogate markers, few predictive biomarkers have been identified to be clinically relevant in a disease-agnostic fashion for prediction of response to immunotherapies in several tumor entities, including the expression of programmed death receptor ligand 1 (PD-L1) on tumor cells, microsatellite instability as well as the mutational burden [3–5]. Moreover, effector T-cells are key players in cancer immunotherapies and have been identified as central prognostic biomarkers in a great number of cancer entities [6, 7]. Accumulating evidence suggests that both CD8^+^ and CD4^+^ T-cells in the tumor microenvironment (TME) play a crucial role in antitumor immune responses, but also that distinct populations may act as predictive biomarkers for immune checkpoint inhibitors [8, 9]. However, present biomarkers are often not sufficient to safely distinct between responders and non-responders, and heterogeneity between different metastatic lesions may contribute to this [10, 11]. Therefore, understanding the complex nature of immune responses within the tumor microenvironment will likely be necessary to improve the definition of single and combined predictive biomarkers for treatment responses and prognosis of patients.

**Fig. 1:**
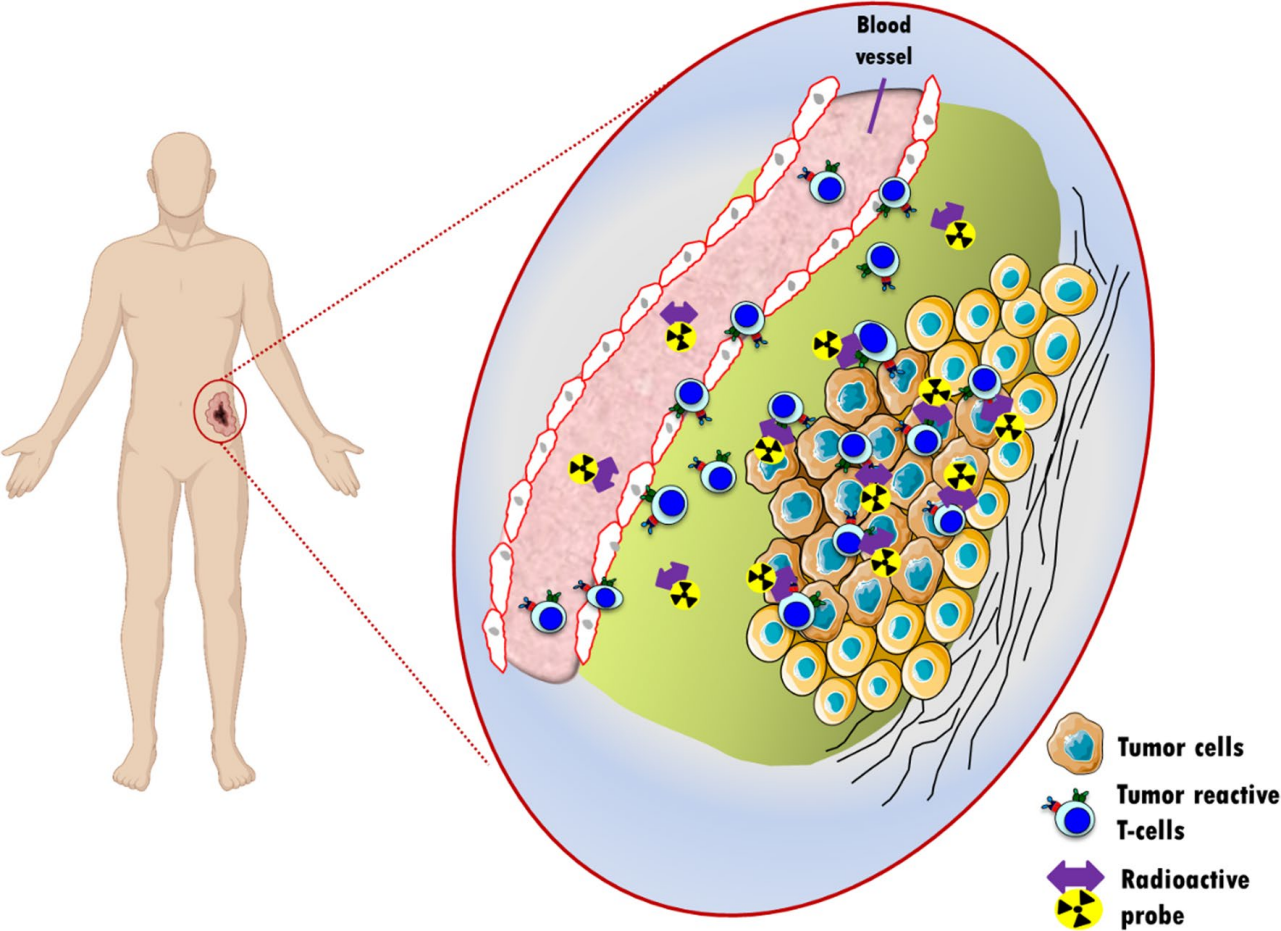
Principle of non-invasive in vivo imaging of lymphocytes trafficking to and into the tumor. Activated tumor-reactive T-cells are intravenously injected into a patient having a tumor specifically recognized by those T-cells. The accumulation of the T-cells within the tumor is visualized in-situ using radiolabelled probes recognizing specific markers expressed on the T-cell membrane. The radioactive probe targeting T-cells can be either a full-size antibody and its derivatives, or a short peptide radiolabelled with a radioisotope (PET or SPECT isotope) matching the plasma half-life of the probe

Imaging technologies are well-established tools for the evaluation of a diversity of treatment strategies. Magnetic resonance imaging (MRI), anatomical imaging by computer tomography (CT) or functional imaging using positron emission tomography (PET) utilizing the properties of 2-deoxy-2-[^18^F]-fluoro-D-glucose ([^18^F]FDG) to determine metabolic activity are currently used as surrogate markers of therapeutic response [12]. While these techniques provide high temporal and spatial resolution, they are not able to accurately depict the complex patterns of tumor immune environment as well as response during immunotherapy, which can differ considerably from traditional therapies [13, 14]. With the field of immunotherapies rapidly evolving, novel imaging technologies may become one of the most crucial surrogate markers in oncology and their development is intensively ongoing. Although a number of different approaches are currently evaluated in preclinical studies [15, 16], nuclear imaging represents an attractive strategy to investigate immune responses including the immune checkpoint axes and the presence of T-cells by targeting defined surface markers (Fig. 1).

## Principles of immuno-receptor and peptide-based tracer development

**Fig. 2:**
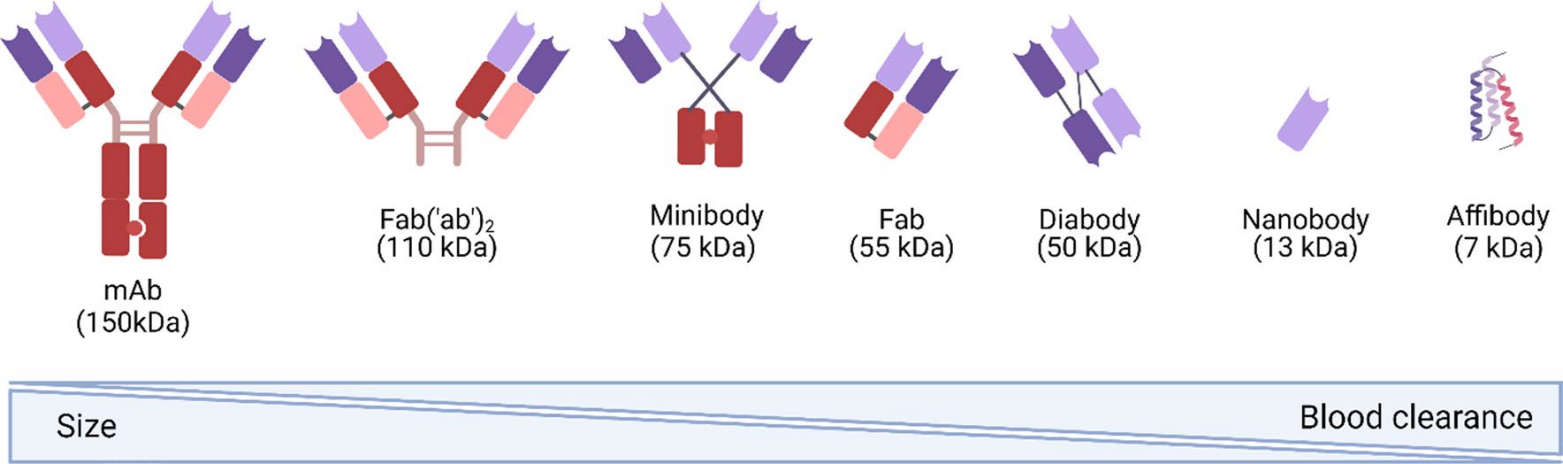
Protein-based constructs used for ImmunoPET and T-cell tracking. This panel points out how the molecular weight (MW) of the different antibody- and peptide-derived probes influence their blood pool clearance, which influences the selection of the right radioisotope for immunoPET

In the past decade, the importance of immunoPET in molecular imaging has increased due to the combination of high target specificity of monoclonal antibodies (mAbs) and the inherent high sensitivity and resolution of the PET-based imaging. ImmunoPET has shown to play a key role as an imaging technique in the field of immunotherapy to deploy host immune system behaviour, to select patients who could potentially benefit of immunotherapy, and to monitor treatment response [17]. For this purpose, protein scaffold molecules that vary in terms of their molecular weight and blood clearance have been investigated for their potential as tools to monitor T-cell distribution and homing in vivo (Fig. 2).

Full size monoclonal (mAbs) have been extensively investigated as probes to target and track immune cell distribution. For example, an anti-mouse CD3 antibody, (clone 17A2; R&D System) radiolabelled with the positron emitter zirconium-89 (^89^Zr), has been investigated as a probe to visualize cytotoxic T-lymphocyte infiltration in preclinical models of colorectal cancer. This probe showed a strong correlation between tumor signal and the number of immune cells at the tumor site [18]. Examples of antibody-derived probes are F(ab′)_2_ fragments generated via enzymatic digestion from full size murine anti-human monoclonal antibody targeting CD2 and CD7 markers expressed on the surface of T central memory cells (T_CM_) and radiolabelled with ^89^Zr [19]. These antibody fragments showed PET signals correlating with tumor infiltrating T_CM_ cells and T-cell anti-tumor efficacy in tumor myeloma preclinical models.

In an effort to further improve the pharmacokinetics of probes used for immunoPET, bivalent minibodies with a molecular weight of 75 kDa have been adapted for tracer-development. While minibodies are only half the size of full mAb and have shown strong tumor accumulation [20], their size and the subsequent relatively slow blood clearance kinetics still result in maximum tumor-uptake days post injection, leading to a high degree of radiation exposure for patients.

Cys-Diabodies (cDb) are one of the conventional engineered antibody fragments used to enhance imaging characteristics, such as rapid clearance for high target-to-background images at short times after injection, reduced radiation dose, engineered sites for site-specific conjugation, and the removal of Fc effector functions, among others [21, 22]. The 169cDb has being appropriately labelled with ^89^Zr and the resulting tracer, [^89^Zr]Zr-malDFO-169cDb, has being reported to be effective for the non-invasive immuno-PET tracking of endogenous CD8^+^ T-cells [23]. The same 169cDb was also labelled with copper-64 (Cu-64) with the aim to accurately visualize and quantify changes in tumor-infiltrating CD8^+^ T-cells in response to immunotherapy [24]. However, as for all protein scaffold constructs above 40 kDa, an aspect called enhanced permeability and retention (EPR) effect can influence tracer binding and may result in false positive results [25, 26].

Nanobodies, the variable domains of heavy chain antibodies (VHHs), are a small antigen-binding derivatives with molecular weight around 15 kDa, high affinity, strong stability, low immunogenicity, fast clearance, and strong tissue penetration [27]. With regards of their application in ImmunoPET, their use for tracking T-cells has been increased by targeting different antigens (Table 1). In 2017 Rashidian et al. used a nanobody-based tracer, ^89^Zr-labelled PEGylated single-domain antibody fragments specific for CD8, to track the presence of intratumoral CD8^+^ T-cells in the immunotherapy-susceptible B16 melanoma model in response to checkpoint blockade. The radiotracer has shown to be able to detect thymus and secondary lymphoid structures as well as intratumoral CD8^+^ T-cells [28]. More recently, a group of nanobodies were studied to be used as an attractive non-invasive tool to discriminate between both systemic and tumor-infiltrating CD8^+^ T lymphocytes. Therefore, the tracer [^68^Ga]Ga-NOTA-SNA006 was selected to precisely track human CD8^+^ T-cells in different mice models, showing great potential for immunotherapy monitoring and efficacy evaluation [29].

**Table 1:** Summary of salient properties of immune and peptide-associated tracers and radioisotopes matching their half-life

	IgG	F(ab′)_2_	Fab′	Diabody	scFv	Nanobodies and affibodies
Molecular weight	150 kDa	110 kDa	55 kDa	40–50 kDa	28 kDa	13–16 kDa
Biol. T_1/2_ blood (h)	110	48	4	< 4	1	< 1
Metabolic target organ	Liver	Liver	Kidney	Kidney	Kidney	Kidney
Optimal accumulation time	Days	Day	Hours	Hours	Hour	< Hour
Radionuclides of interest for PET	64Cu 89Zr	64Cu 89Zr	64Cu 18F	68Ga 18F	68Ga 18F	68Ga 18F
Half-life	12.7 h 78.4 h	12.7 h 78.4 h	12.7 h 110 min	68 min 110 min	68 min 110 min	68 min 110 min
E_max_β + (MEV)	0.653 0.902	0.653 0.902	0.653 0.634	1.890 0.634	1.890 0.634	1.890 0.634
Branching (β+) %	17.5 22.7	17.5 22.7	17.5 96.9	87.7 96.9	87.7 96.9	87.7 96.9
Intrinsic spatial resolution loss (mm)	0.7 1.0	0.7 1.0	0.7 0.7	2.4 0.7	2.4 0.7	2.4 0.7

The smallest protein scaffolds used in immunoPET, which have been primarily used for cancer-diagnosis and now are being investigated in immuno-imaging, are affibodies. Affibodies (dimers of 6–7 kDa) are engineered as small, robust (heat-resistent up to 90 °C [30]) and fast-clearing [31] biomolecules, which have already shown great potential for the diagnosis of cancer. In a phase I clinical trial, a HER2-targeting ^111^In-labelled affibody was shown to be safe in humans and to allow the visualization of breast cancer metastases (e.g. in brain and in lymph nodes) at 4–24 h post tracer injection via SPECT/CT scan [32]. Despite the advantages gained with their low molecular weight and their fast clearence for imaging, affibodies have to overcome certain obstacles such as decreased avidity for their respective targets [33] and the possibility of increased lipophilicity after labelling, resulting in increased off-target interactions [34].

The major challenge in developing imaging tools using protein scaffolds is to match their size (MW) and their blood half-life with radioisotopes matching their body distribution (Fig. 2).

### Imaging of immune checkpoint axes

Immune checkpoint modulation, mostly antibody-based therapeutics, has opened new possibilities in the context of cancer treatment. Although immune checkpoint inhibitors have markedly improved patient’s survival, this benefit is mainly limited to a minority subpopulation that achieves a response. Therefore, predicting which patients are most likely to benefit of immunotherapy would be valuable for individual therapy optimization [35]. In particular, using radiolabelled immune checkpoint inhibitors (ICIs), it may be possible to investigate the potential responsiveness to this therapeutic approach or to directly image infiltrating tumor reactive T-cells expressing these immune checkpoints. Tumor cells upregulate PD-L1, which binds PD-1 expressed on T-cells. PD-1/PD-L1 interaction reduces the activity, proliferation and survival of T-cells [36]. The use of antibodies targeting this axis has already provided important results. Being able to perform quantitative imaging of the in vivo distribution of these therapeutic antibodies can represent a great step forward in the selection of patient, optimization of treatment schedules and design of novel combination therapies. The feasibility of this new “theranostic approach” has been demonstrated in preclinical studies, for example using ^64^Cu-labelled anti-PD1 antibody showing tracer accumulation via PET imaging in both lymphoid organs and tumor [37, 38]. A first-in-human clinical application of this approach exploited ^89^Zr-labelled nivolumab in non-small cell lung cancer patients to assess PD-1 expression in the tumor prior to anti-PD-1 treatment by Niemeijer and colleagues. [^89^Zr]Zr-DFO-nivolumab uptake pre-treatment was higher in responding tumor lesions as compared to non-responding tumors, with a higher predictive score than gold-standard immunohistochemical markers. Along the same line, two studies on [^89^Zr]Zr-DFO-pembrolizumab imaging (NCT03065764, NCT02760225) are currently open for locally advanced, metastatic melanoma or non-small cell lung cancer [39].

Another checkpoint inhibitor is the cytotoxic T lymphocyte-associated antigen 4 (CTLA-4). By targeting this checkpoint expressed on activated T-cells using a specific antibody, it is possible to substantially enhance anti-tumor activity [40, 41]. In order to understand the expression level of CTLA-4 on activated T-cells infiltrating tumor, an anti-CTLA-4 monoclonal antibody was radiolabeled with ^64^Cu ([^64^Cu]Cu-DOTA-anti-CTLA-4) and its binding specificity tested in vivo in CT26 tumor bearing mice. In this study, Higashikawa and colleagues showed a high tracer uptake on T-cells and not on the tumor, and correlated it with the high expression of CTLA-4. Based on that, a phase 1 clinical trial with [^89^Zr]Zr-DFO-ipilimumab in metastatic melanoma patients was started [42]. Lymphocyte activation gene-3 (LAG-3), a CD4-like molecule belonging to the immunoglobulin superfamily expressed on activated CD4^+^ and CD8^+^ T-cells and other immune cells subpopulations, is one of the most studied next-generation immune checkpoints. It has been investigated to compensate for the loss of response to CTLA-4 and PD-1/PD-L1 mAbs in subsets of patients, and as a target for the treatment of cancer patients [43–47]. Relatlimab was the first anti-LAG-3 mAb that entered clinical testing, as a mono- or combination therapy with nivolumab, in melanoma, renal cell carcinoma and non-small cell lung carcinoma (NSCLC) (NCT019680109), and it showed to restored T-cell functionality [48]. To take advantage of favorable pharmacokinetics and tumor penetration compared to a full monoclonal antibody (mAb), a nanobody targeting LAG-3 was developed to specifically target and allow quantification of LAG-3 expression on tumor-infiltrating lymphocytes (TILs) in different mouse cancer models treated with anti-PD-1 mAbs [49]. Thus, nanobodies targeting this checkpoint showed to be an interesting diagnostic tools for noninvasive detection of LAG-3 before and after ICI-treatment.

### Direct imaging of T-cells

Monitoring temporal distribution, homing dynamics and anti-tumor responses of T-cells in vivo is an essential step during optimization of T-cell-based immunotherapies. Visualizing the T-cell response can be based on a variety of targets, that come with their own set of advantages and applications.

**Fig. 3:**
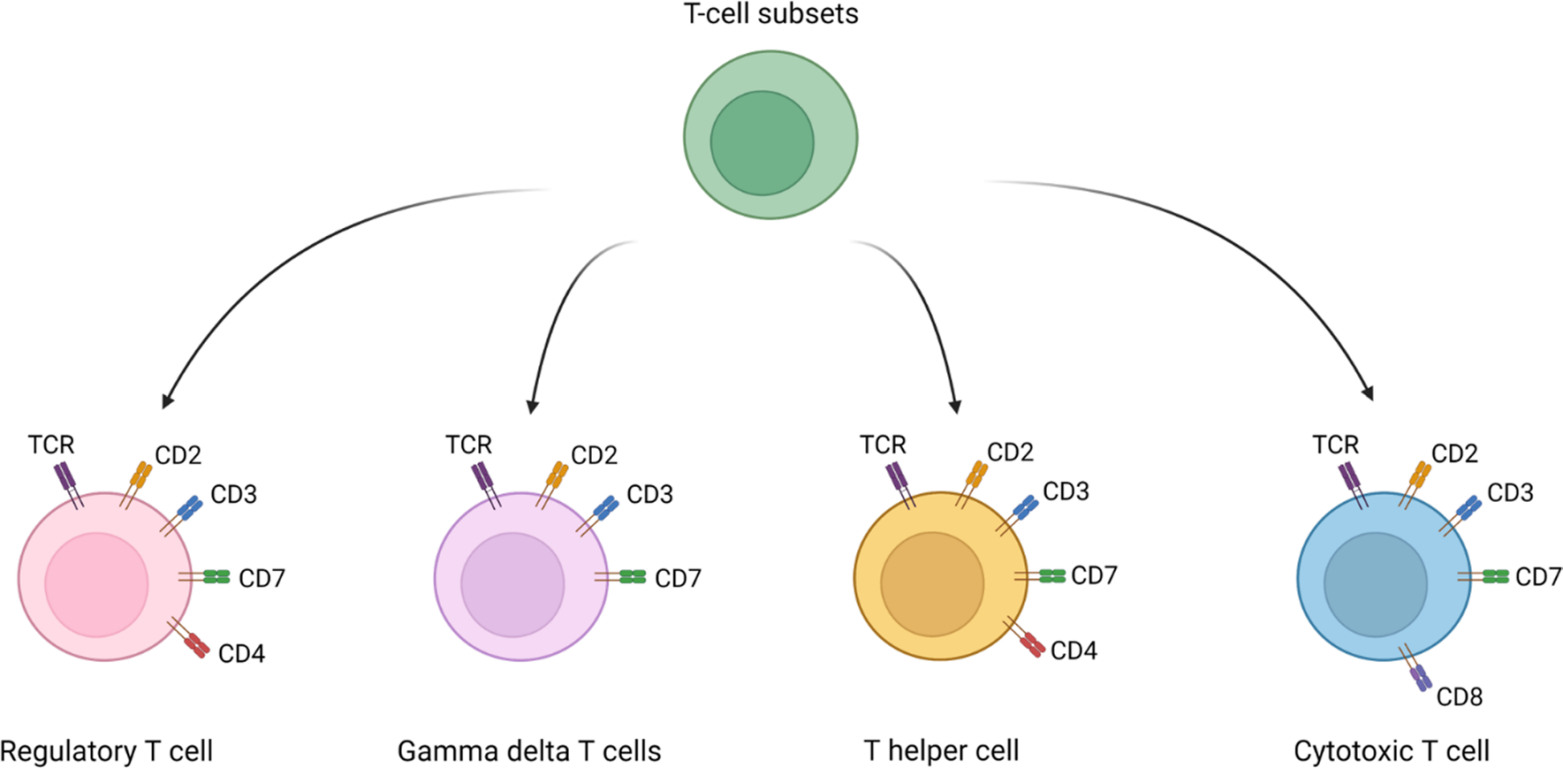
Differentiation of T-cell subsets and their respective cell surface marker

A number of diverse surface markers have been investigated for their suitability so serve as target for immunoimaging approaches including CD4^+^ and CD8^+^ T-cell subpopulations, but also pan-T-cell markers (Fig. 3). Besides their expression characteristics, it is crucial that targeting one of these markers does not modulate the properties of the corresponding T-cell in any way but instead acts as an inert T-cell marker, which we will discuss in detail later on in this review.

CD3 is a highly specific cell surface molecule on T-cells, which acts as a co-receptor for the T-cell receptor (TCR) during immune responses [50]. During the antigen recognition by the TCR, interaction with its CD3 co-receptor leads to T-cell stimulation and activation [51]. CD3-targeting imaging approaches have been successfully tested in preclinical models [52]. Larimer and colleagues have shown a high level of infiltration of T-cells during anti-CTLA4 treatment by targeting the T-cell surface glycoprotein CD3 in colon cancer xenograft models using a murine ^89^Zr-labelled anti-CD3 [18]. However, antibodies targeting the CD3 domain are potentially influencing and modulating the T-cell function, which is dependent on the specific CD3 antibody and the used dose [53]. In fact, it has been also demonstrated that distinct concentrations are capable of not only stimulating T-cell proliferation but also increasing IFNγ production, indicating an enhanced state of T-cell activation [19, 54]. While the expression profile of CD3 would qualify it as an attractive pan T-cell marker, its modulatory effects on T-cell functionality may be problematic in case of diagnostic T-cell tracking.

As a member of the immunoglobulin superfamily, CD7 is a known marker for mature T-cells, Natural Killer (NK) cells and early-stage hematopoietic precursor cells [55]. The expression of CD7 on hematopoietic precursor cells, which can potentially differentiate into B cells or myeloid cells, may represent a limitation for T-cell tracking. However, since both B cells and myeloid cells lose their CD7 expression during maturation, high CD7 expression on immune cells remains specific to T-cells. CD7 targeting F(ab′)_2_ has already successfully been used in preclinical in vivo studies to monitor T-cells in the context of adoptive T-cell therapy [19]. Mayer & Mall et al. showed that PET imaging using the zirconium-89-labelled anti-CD7 F(ab′)_2_ provided a strong signal at the tumor site while having no long-term impact on T-cell functionality in vivo. Intravenous injection of anti-CD7 F(ab′)_2_ did not modulate T-cell functionality in vivo and tumor rejection was unaffected, making CD7 a promising target for inert and non-invasive T-cell tracking across the immunotherapeutic spectrum.

An alternative target is CD2 which is expressed on T-cells [56], NK cells [57] and thymocytes [58]. What makes it interesting is the high correlation of its expression with cytolytic activity in tumors, suggesting CD2 as membrane marker to track effector T-cells [59]. Mayer et al. selected several antibody clones targeting CD2 from which radiolabelled derivatives (^89^Zr-labelled F(ab′)_2_) were produced and used for T-cell tracking, because of their general superiority with respect to in vivo pharmacokinetics compared to full size antibody, and because of absence of functional antitumor efficacy impairment of targeted T-cells in vitro [19]. Further investigation in vivo via PET/CT imaging, using a previously described mouse model of adoptive T-cell transfer, resulted in a very distinct signal at the tumor site and high contrast images [60]. However, the impact of the radiotracer on T-cell functionality in vivo demonstrated anti-CD2 F(ab′)_2_ to induce severe T-cell depletion and failure of tumor rejection.

Targeting the T-cell receptors (TCR) allows for specific tracking of genetically modified T-cells during adoptive T-cell transfer and serves as a tool to optimize T-cell therapy and to understand pharmacodynamics and pharmacokinetics of TCR-modified T-cells. Constant membrane turnover of TCRs allows for effective labelling, resulting in fast and constant internalization. Using this principle, an anti-chicken OVA-TCR antibody (TCR DO11.10) was labelled with ^64^Cu and successfully internalized within 24 h of TCR-mAb binding [61]. Antigen recognition remained stable after labelling and neither viability, nor DNA-damage or induced apoptosis were negatively affected while still yielding high contrast PET images. While this approach cannot be applied in a clinical setting due to specificity for a defined murine OVA-specific TCR, murinization of the constant domain of TCR moieties as general optimization strategy for transgenic human TCR may lead to the detection of TCR-transduced human T-cells by either an anti-murine TCR monoclonal antibody or fragment derivatives (F(ab′), F(ab′)_2_) [62]. The feasibility of this approach has been investigated, showing the potential as highly sensitive tool for mapping TCR-transgenic T-cells within a xenogenic human myeloid sarcoma mouse model, using an antiTCRmu F(ab′)_2_-fragment radiolabelled via zirconium-89 methodology and then used to perform a small animal PET/CT [32]. However, immune imaging strategies targeting murine sequences of the TCR are limited to the scope of monitoring TCR-engineered T cells, while excluding endogenous T-cells in the context of other immunotherapies. This limitation is equally present using other reporter genes, which have shown to be effective for the tracking of genetically modified T-cells in vivo [63, 64].

Most imaging approaches to track T cells in vivo, however, currently focus on visualizing CD8^+^ T-cell subpopulations [24]. The reason for this is the well-documented fact that tumor-infiltrating CD8^+^ T cells play an essential role during the anti-tumor response, and that they have been shown to correlate with an improved prognosis for tumor entities such as non-small cell lung cancer (NSCLC) [65], colorectal cancer [66], ovarian cancer [67] and melanoma [68]. Furthermore, in the context of immunotherapy, it was shown that patients with a high degree of pre-existing and/or tumor infiltrating CD8^+^ T cells were more likely to respond well to PD-1/PD-L1 immune checkpoint inhibitors [69–71]. Therefore, a thorough understanding of the localization and dynamics of CD8^+^ T cells in vivo is considered important for prognosis and evaluation of immunotherapy patients.

Since CD8^+^ T cells were an early focus of T-cell imaging, preclinical research of this topic is well established, and there are numerous approaches to depict the CD8^+^ T-cell population. One study was utilizing an anti-CD8 cys-diabody labelled with ^89^Zr to help monitoring endogenous CD8^+^ T cells in the context of antigen-specific adoptive T-cell transfer, agonistic antibody therapy as well as checkpoint blockade antibody therapy [23]. While this approach comes with the inherent limitations of both the relatively large minibody-construct as well as the long-lived positron emitter ^89^Zr, another approach tries to improve both aspects by utilizing a ^68^Ga-labelled anti-CD8 nanobody [29]. By using a fast blood-cleared nanobody combined with ^68^Ga and its half-life of 68 min, this approach aims to minimize the organ radiation exposure and consequential side effects paid for by the patient. However, detailed analysis of potential effects on T-cell functionality after anti-CD8 nanobody binding are missing, even though essential when aiming for clinical translation.

As reported, initial studies are promising and suggest that CD8-based PET-imaging in patients may be feasible, and has the potential to depict a very important part of the human immune-response. However, further validation is required and monitoring only the CD8^+^ population may fail to depict the multifaceted aspect of T-cell responses during immunotherapy. For instance, recent advantages have shown that CD4^+^ T-cells play a dominant role in anti-tumor response and are associated with response patterns of immune checkpoint therapy [72, 73]. Furthermore, recent advances in CAR-T cell therapy have highlighted the importance for a widespread and encompassing evaluation and monitoring of T-cell responses. CD4^+^ CAR-T cells have shown great promise in leukemia patients, where CD4^+^-, not CD8^+^ CAR- T cell treatment led to long-term tumor eradication [74].

Despite the huge number of tracers developed and tested in preclinical studies to illuminate the fate of activated T-cells during immunotherapy, to date none of these tracers for T-cell tracking has been approved by either the FDA or the EMA. However, in the last decades, some approaches have been tested in pilot studies and entered clinical trials, and are currently being investigated for their clinical potential. One approach is the tracking of CD8 positive T-cells using an ^89^Zr-labelled anti-CD8 minibody in the context of patients with solid malignancies (NCT03107663). Preliminary data showed that the CD8 tracer did not induce any immediate or delayed side effects, and that the highest uptake of the ~ 55 kDa sized minibody was seen in spleen and bone marrow, with a low kidney accumulation [75]. Two patients, one affected by melanoma and one by hepatocellular carcinoma, showed a distinct metastatic lesion uptake as early as 2 h post injection, with the highest uptake in most lesions at 24 h or 48 h post injection. The remaining four patients, all suffering from lung cancer, did not show significant uptake of anti-CD8 minibody in their respective lung metastases. Furthermore, the tracer showed favorable pharmacokinetics, allowing for early imaging as soon as 6 h to 24 h post injection. However, no evaluation of potential effects of the tracer on T-cell viability, -proliferation and -function in vivo have been reported. In addition, the reported whole-body clearance of the used minibody was similar to a full-size antibody [76]. Thus, the radiation exposure of the patient per MBq injected activity can be expected to be at least 10-times higher than of a FDG PET/CT scan, thereby potentially limiting the clinical feasibility and utility of this imaging agent.

## Imaging of ex vivo labelled T-cells

**Fig. 4:**
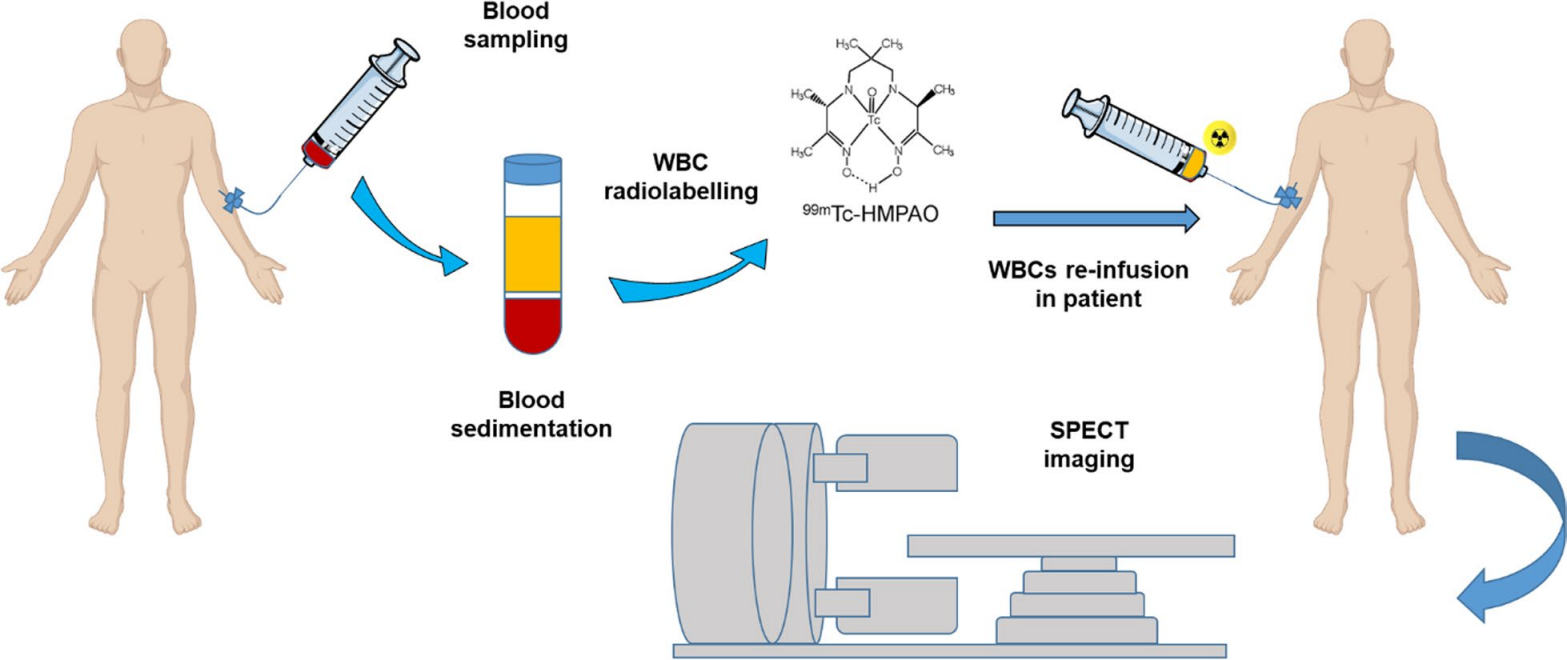
Schematic representation of direct T-cell labelling imaging approach. After blood sampling, white blood cells (WBCs) are isolated from peripheral blood mononuclear cells (PBMCs) via density-gradient centrifugation, and incubated with [^99m^Tc]Tc-HMPAO (shown in the panel) or alternatively with [^111^In]In-oxine. The radiolabelled WBCs are then re-infused in patients and their homing in inflamed organs visualized via single photon emission tomography (SPECT) imaging

An alternative approach to track T-cells via targeting surface-bound markers is the ex vivo radiolabelling of isolated white blood cells (WBC) using [^111^In]In-oxine or [^99m^Tc]Tc-hexamethylpropylene amine oxine ([^99m^Tc]Tc-HMPAO) (Fig. 4). These radiotracers pass through the plasma membrane of WBCs isolated from peripheral blood mononuclear cells (PBMSc) via passive transport, and accumulate in the cytoplasm. Radiolabeled WBCs are re-injected into the patients and used to localize inflammatory and infectious conditions, or tumor infiltrating lymphocytes.

Early clinical studies used [^111^In]In-oxine labelling of CD4^+^ T-cells to investigate the homing of CD4^+^ T-cells in Hodgkin’s lymphoma lesions with SPECT imaging, and accumulation of adoptively transferred tumor infiltrating lymphocytes, previously expanded ex vivo, in melanoma patients [77, 78]. [^99m^Tc]Tc-HMPAO is nowadays used as standard methodology for labelling autologous leukocytes due to better image quality compared to ^111^In, and isotope availability. However, if not completely reduced intracellularly, [^99m^Tc]Tc-HMPAO may be released from cells with time, especially in those patients affected by metabolic dis-functions [79]. Radiolabelled WBCs methodology has been successfully applied, for example, to assess intestinal lymphocytes infiltration in Crohn’s disease (CD), allowing discrimination between intestinal and bone marrow uptake within the pelvis area [80]. Furthermore, the increased understanding and subsequent significance of gammadelta T-cell therapy has led to promising ex vivo imaging approaches using [^89^Zr]Zr(oxinate)_4_ as a cell-labelling agent for therapeutic gammadelta-T cells, allowing for specific and clinically applicable PET imaging [81]. Disadvantage of the direct labelling approach is the need of highly trained personal with experience in good manufacturing practice (GMP), as well as cell handling at the treatment side. In addition, with respect to track endogenously stimulated T-cells, tumor-specific T-cells circulating in the peripheral blood may have low frequencies and migration of blood-derived labelled cells into the tumor is so far ill-defined [82].

## Peptide-based probes to image immune effector functions

Lymphocytes may also be imaged by targeting cell functional markers for cytokine secretion. Interleukin-2 (IL-2) has been shown to stimulate in vitro the generation of cytotoxic T lymphocytes and of lymphokine-activated killer cells, both of which are involved in killing of cancer cells. For this reason, a radiolabelled version of IL-2 ([^99m^Tc]Tc-IL2) has been developed and used to detect in vivo the presence of activated organ-infiltrating lymphocytes, and in particular those expressing cluster designation (CD) 25 antigen (IL-2 receptor [IL2R]) in melanoma patients undergoing surgical treatment [83]. In this preliminary study, a good correlation between scintigraphy signal and extent of tumor infiltration of IL2R-positive cells was found. To facilitate the routine production of ^99m^Tc-labelled IL-2 and clinical application of this tracer, a single-step radiolabelling approach was developed and optimized to produce [^99m^Tc]Tc-HYNIC-IL2 [84]. In a pilot study, patients with metastatic melanoma receiving ipilimumab or pembrolizumab were subjected to SPECT/CT imaging with [^99m^Tc]Tc-HYNIC-IL2 with the aim to detect tumor infiltrating lymphocytes (TILs) [85]. In 5 patients (2 treated with ipilimumab and 3 with pembrolizumab), metastatic lesions could be visualized with a positive correlation between size of the tumor lesion and [^99m^Tc]Tc-HYNIC-IL2 accumulation, both before and after 12 weeks of therapy. Upon immunotherapy, some lesions showed increased uptake, whereas other lesions demonstrated decrease. The limited number of patients and availability of histological validation did not allow to draw conclusions on potential relation between the numbers of tumor-infiltrating lymphocytes and level of [^99m^Tc]Tc-HYNIC-IL-2 uptake. In addition, due to the fact that IL-2 is biologically active, low amount of radiolabeled IL-2 can be injected in patient, representing this the major drawback for using this tracer to evaluate the severity and extend of peri-tumoral or intra-tumoral lymphocytic infiltration, in particular via SPECT imaging.

To overcome the poor sensitivity and spatial resolution of SPECT imaging, a ^18^F-labelled IL-2 tracer has been developed via conjugation of IL-2 with succinimidyl 4-[^18^F]-fluorobenzoate ([^18^F]SFB) for PET imaging [86]. An increase in N-(4-[^18^F]fluorobenzoyl)-interleukin-2 ([^18^F]FB-IL-2) uptake in mice models when tumors were either irradiated or immunized was shown, and further increase in tumor tracer accumulation was observed when treatment were combined, indicating a synergistic effect. On the other hand, the use of a CXCR4 antagonist induced inhibition of tumor cell infiltration, followed by decrease in tracer uptake, indicating that ([^18^F]FB-IL-2) may guide cancer immunotherapy. [^18^F]FB-IL-2 is currently being investigated in clinical trials (EudraCT: 2014–003,387.20). Unfortunately, in the preliminary study carried out by the same group in patients affected by metastatic melanoma and injected with 200 MBq fluorine-^18^F-labelled IL-2, a correlation between treatment-related immune response and tracer accumulation was not detected [87]. This result is maybe due to the low number of patients enrolled in the study and necessitate to be confirmed with a large cohort of patients. On the other hand, this is a confirmation of the difficulty to translate pre-clinical results obtained using animal models to clinical application in the context of cancer immunotherapy.

Other effector molecules associated with inflammatory anti-tumor immune responses have been explored as imaging targets, with the aim to increase specificity in the detection of effective immune response, rather than increased presence of T-cells. Among these, granzyme B has been studied as indicator of anti-tumor T-cell function and marker for PET imaging during immunotherapy. Granzyme B is a serine-protease released by CD8^+^ T-cells [88] and NK cells [89] during cellular immune response. To identify patient responders to cancer immunotherapy, Larimer and colleagues designed a molecular tracer starting from the cleavage sequence of murine granzyme B, modified it with NOTA chelator and radiolabelled with gallium-68 to produce a granzyme B specific PET agent (GZP) [90]. In particular, to assess the translational potential, tumor biopsies from patients subjected to immunotherapy were used to establish syngeneic colon cancer models to target granzyme B expression using a peptide-based probe ([^68^Ga]Ga-NOTA-GZP). This probe showed to be able to distinguish between responders and non-responders during therapy, with a correlation between PET signals and granzyme B expression in tumor samples [90, 91].

## Radionuclides used for T-cell nuclear imaging

The use of radioisotopes in both SPECT and PET diagnostic modalities should be properly selected based on several characteristics like their half-life, decay modality and energy, as well as their availability. For diagnostic purposes, a radionuclide with relatively limited energy (100–200 keV) and a high average path (typical γ rays) that can be detected by a detector near the patient is required. The half-life of a radionuclide should be then short for imaging purposes, and the production should happen possibly locally. Furthermore, after the decay, the nuclide should lead to a low activity isotope that can be easily excreted from the organism. Examples of radionuclides that suit these requirements are ^68^Ga, ^18^F, ^99m^Tc or ^89^Zr (Table 2).

**Table 2:** Radioisotopes used for in vivo T-cell imaging/tracking and their physical characteristics

	Positron emitter	Half-life	Main β^+^/γ energy (Mev)	β^+^ decay (%)	Intrinsic spatial resolution loss (mm)	Production method
PET	^18^F	109.77 min	0.634	97	0.7	Cyclotron
	^68^Ga	67.71 min	1.899	88	2.4	Generator
	^89^Zr	78.41 h	0.900	23	1.0	Cyclotron
SPECT	^99m^Tc	6.01 h	γ:0.141	IT	4.0	Generator

The most used radioisotopes for T-cell tracking in clinical application are reported. β^+^, positron decay; IT, isomeric transition

The radiolabelling of biomolecules such as full size antibodies, or their derivatives (F(ab′)_2_ and Fab′, nano- and diabody, or peptides can be performed by selecting the best radionuclide for a given application assuring that the physical half-life of the radionuclide matches the expected biological half-life of the vector molecules in vivo [92].

Ga(III) metal has a preference to form 6-coordinate complexes with an octahedral coordination geometry [93]. With regard to the radiolabelling of sensitive biomolecules, Zhao et al. have reported the use of the chelator 1,4,7- triazacyclononane-N,N′,N″-triacetic acid (NOTA) in the design of a specific tracer to track human CD8^+^ T-cells in vivo via ImmunoPET [29]. A nanobody-based radiotracer, namely [^68^Ga]Ga-NOTA-SNA006a, targeting human CD8 antigen was synthesized with high RCP (RadioChemical Purity) and high affinity, showing promising performances in tracking of human CD8^+^ T-cells in mice models, compared with other candidates.

^18^F is normally introduced in the structure of a tracer via indirect fluorination by conventional nucleophilic substitution using a prosthetic group [94, 95], but since this method required harsh conditions not always suitable for sensitive proteins, in the past decade the complexation of [Al^18^F]^2+^ became the most studied methodology as an alternative to standard radiofluorination. This can be done either by conventional chelators such as NOTA and NODA at high temperature, not fitting with heat sensitive molecules (Fab′, scFv, diabody or nanobodies), or by recently restrained complexing agents (RESCA) published by Cleeren et al. and the AMP-based chelators developed by our group [96–98]. The RESCA chelator was then succesfully used for the labelling of interlukine-2 (Al[^18^F]F-RESCA-IL2) as alternative of [^18^F]-FB-IL2 showing good in vitro and in vivo characteristics, with high uptake in lymphoid tissue and hPBMC xenografts [99]. Despite the AlF-18 method being very convenient for the labelling of biomolecules, its application with regard of T-cell tracking is still limited, hence, the most common methodology so far is still the radiofluorination via prostetic group. As already shown above, an example of this proposes interleukin-2 as marker for imaging, as activated T-cells bind higher amounts of IL-2 due to increased expression of IL-2RA (CD25). In this case, IL-2 was labelled with fluorine-18 via reaction with succinimidyl 4-[^18^F]-fluorobenzoate ([^18^F]SFB) to monitor tumorreactive T cells directly at the tumor site [86].

Zirconium-89 (^89^Zr) is a radionuclide with suitable half-life (78.4 h) and energy (0.902 MeV) to match the half-life of radiotracers based on the use of full size antibodies or antibody-derived fragments as targeting vectors, which require long periods (days to weeks) to few hours to fully accumulate at the target site in vivo, and which are of great importance for the use of immune-PET. In 2017, Vugts and colleagues developed a derivative of the gold standard chelator DFO, called DFO* that based on the good stability in vivo and a low accumulation in bones was recently highlighted to be the next candidate for clinical translation [100, 101]. Due to its physical characteristics, Zr-89 has been often used to develop new immunoPET tracers for T-cells trafficking and imaging. To visualize T-cell responses, for example, Larimer and colleagues have shown a high level of infiltration of T-cells during anti-CTLA4 treatment by targeting the T-cell surface glycoprotein CD3 in colon cancer xenograft models using a murine ^89^Zr-labelled anti-CD3 [18]. Lymphocytes-activation gene 3 (LAG3) is another cell surface marker that has been investigated in mice models bearing the human variant of the LAG3 (MC38/hLAG3) by using a fully anti-LAG3 antibody labelled with [^89^Zr]Zr-DFO ([^89^Zr]Zr-REGN3767 with the aim to predict and monitor therapy response with and without anti-PD1 treatment [102]. To get a read out of the slow accumulation of full-size antibodies, in particular in peripheral tissues, engineered antibody fragments, like cys-diabodies or minibodies (see Fig. 2), targeting CD4 and CD8 have been developed and radiolabelled with zirconium-89 ([^89^Zr]Zr-malDFO-GK1.5 cDb and [^89^Zr]Zr-Df-IAB22M2C respectively) [23, 75, 103]. Studies of the clinical feasibility of this approach are ongoing (NCT03802123). Our group has pursued in vivo T-cell imaging by tracking engineered human T-cells using a ^89^Zr-labelled anti mouse TCR F(ab′)_2_ fragment ([^89^Zr]Zr-Df-aTCRmu-F(ab′)_2_), which is selective for the murine TCR beta domain of a transgenic TCR. Using this approach, we have been able to visualise different number of transgenic T-cells injected intravenously, and to correlate the PET signal with the total number of T-cells detected ex vivo independently from the tumor engraftment rate [104].

As already described above, technetium-99m (^99m^Tc) is another radioisotope that has been used for radiolabelling of interleukin-2 modified by conjugation with succinimidyl-6-hydrazinopyridine-3-carboxilate (HYNIC-NHS) as a bifunctional chelating agent and tricine as co-ligand, based on the fact that combination of HYNIC-peptide with tricine produces a ternary ligand system which forms a stable technetium complex [84]. [^99m^Tc]Tc-HYNIC-IL2 has been afterwards tested in clinical studies for evaluating the ability to target IL-2RA (CD25) expression on activated T-cells using SPECT imaging [85]. To overcome the poor sensitivity and spatial resolution of SPECT imaging, an ^18^F-labelled IL-2 tracer for PET imaging may represent an alternative [105].

## Specific challenges of immune imaging in cancer immunotherapy

Since T-cell imaging aims to visualize the key players during the immune response, tracers face a set of special requirements to allow for safe and specific immuno-PET imaging. While tumor-tracers merely should not activate its target structures and can even be used for simultaneous radio-therapy, T-cell tracers used for diagnostic purposes should not impact T-cell function in any way [106]. Investigating a potential modulating effect is crucial especially in the context of immuno-PET, as the defined monoclonal antibodies and its derivatives can have depleting effects on T-cells and other immune cells with immunotherapeutic relevance [107, 108]. Besides radionuclide-based impairments of T-cell function, which we discuss below, binding of the respective target antigens themselves can have large implications for tracer-suitability. There are a number of publications demonstrating a potential hazard which needs to be taken into account. An anti-murine CD4 cys-diabody showed a dose dependent restriction of both T-cell proliferation and IFNγ secretion in vitro, and subsequent in vivo experiments showed that small doses of the tracer led to reduced proliferation of cells in the inguinal lymph nodes [103]. Modulation of T-cell function that might seem advantageous at first can also have severe consequences as dose-dependency of effects are difficult to predict in vivo. For instance, an increase in cytokine secretion after tracer binding does not necessarily correlate with an accelerated tumor rejection, but can lead to a failed tumor rejection which maybe mediated by T-cell overstimulation and cell death [19]. Other risks potentially associated to over-activation of the T-cells are cytokine release syndromes (CRS) [109]. Furthermore, the antigen-specific domain of tracers can have a significant impact on T-cell function as well. A monoclonal antibody targeted against the murine domain of a TCR (TCRmu), which was introduced in human T-cells, did induce a dose-dependent increase of cell apoptosis and IFNγ secretion after binding of the aTCRmu-IgG [60]. However, a F(ab′)_2_ fragment targeting the same TCRmu structure did not show any alterations on the function of the targeted T-cells, indicating that not just the target antigen is crucial to secure the inert property of the tracer, but also the targeting structure or size. Combined, these aspects stress the importance of comprehensive assessment of T-cell function for T-cell tracer development.

Similar to target and antibody construct-associated effects, the impact of radioactivity on T-cells damage may be of concern. It has been reported, that lymphocytes subsets differ in radiation sensitivity. In particular, lethal irradiation for bone marrow preconditioning does not eliminate all lymphocytes equally [110]. In very general terms, a spectrum of radiosensitivity exists from B cells through naïve T-cells, NK cells, towards more radioresistant T memory cells, NKT-cells, and Tregs [111–113]. There is a tendency towards apoptosis denoting a more radiosensitive phenotype, with activated lymphocytes that are more radioresistant [114]. For example, the effect of radioactivity on memory T-cells imaged using radiolabelled antibody fragments was studied both in vitro and in vivo by Yusufi and colleagues [104]. The exposure of T-cells to a ^89^Zr-labelled tracer was investigated in vitro with a closer look on DNA damage and viability of T-cells by measuring the expression of nuclear DSB-marker γH2AX. TCR-transduced T_CM_ cells were exposed to different activities of [^89^Zr]Zr-Df-aTCRmu-F(ab′)_2_ up to 37 MBq showing that the tracer leads to double-strand breaks (DSBs) and cell death only at higher activities not clinically meaningful. Furthermore, the possible damage of T-cells, once translated in vivo, was also assessed by immunohistochemical (IHC) analysis for CD3, γH2AX and cleaved Caspase-3 after ex vivo biodistribution 48 h p.i. of 2.2 MBq of [^89^Zr]Zr-Df-aTCRmu-F(ab′)_2_ tracer. A high number of tumor-infiltrating T-cells was detected on tissue-level and while the tumor cells expressed γH2AX and cleaved Caspase-3 at high activities, no enhanced DSB were observed within the tumor-infiltrating T-cells indicating no depletion of T-cells by the ^89^Zr-based tracer also in vivo. Further studies are needed to understand the impact of the irradiation on diverse T-cell subpopulations. Taken together, tracers used for T-cell imaging have to be thoroughly characterized both in terms of their target structure as well as the used radioisotope to facilitate their clinical use without non-essential risks for the patient.

## Conclusion

In this review we provide an overview on the more promising tools already available for imaging and tracking of reactive T-cells during immunotherapy, tested both in preclinical and also in clinical application. There is increasing pressure for optimized tracers to assess the success of immunotherapies, including genetically engineered T-cells, therefore we put an accent on the challenges that must be faced and taken into account for clinical implementation of these new tools. In particular, due to the fact that by T-cell tracking we are monitoring the distribution and homing of a living target, during the design of new probes one has to take into account the pharmacokinetic of the probe and blood pool clearance, as well as the specificity of the tracer. This is definitely of great importance, for example, in the case of antibody derived-based tracers, and it influences directly the choice of radioisotope used for tracking and visualizing tumor-reactive T-cells accumulation. Another challenge is represented by the need to use a tracer that do not impair T-cell functionality or do not present biological effects. All in all, the field of T-cell tracking and imaging is an intriguing and hot topic for researchers in the era of immunotherapy.

## Data Availability

Not applicable.

## References

[1] Waldman AD, Fritz JM, Lenardo MJ (2020). A guide to cancer immunotherapy: from T cell basic science to clinical practice. Nat Rev Immunol.

[2] Chiou VL, Burotto M (2015). Pseudoprogression and immune-related response in solid tumors. J Clin Oncol.

[3] Davis AA, Patel VG (2019). The role of PD-L1 expression as a predictive biomarker: an analysis of all US Food and Drug Administration (FDA) approvals of immune checkpoint inhibitors. J Immunother Cancer.

[4] Luchini C, Bibeau F, Ligtenberg MJL, Singh N, Nottegar A, Bosse T (2019). ESMO recommendations on microsatellite instability testing for immunotherapy in cancer, and its relationship with PD-1/PD-L1 expression and tumour mutational burden: a systematic review-based approach. Ann Oncol.

[5] Samstein RM, Lee CH, Shoushtari AN, Hellmann MD, Shen R, Janjigian YY (2019). Tumor mutational load predicts survival after immunotherapy across multiple cancer types. Nat Genet.

[6] Angell HK, Bruni D, Barrett JC, Herbst R, Galon J (2020). The immunoscore: colon cancer and beyond. Clin Cancer Res.

[7] Linette GP, Carreno BM (2019). Tumor-infiltrating lymphocytes in the checkpoint inhibitor era. Curr Hematol Malig Rep.

[8] Wei SC, Levine JH, Cogdill AP, Zhao Y, Anang NAS, Andrews MC (2017). Distinct cellular mechanisms underlie anti-CTLA-4 and anti-PD-1 checkpoint blockade. Cell..

[9] Thommen DS, Koelzer VH, Herzig P, Roller A, Trefny M, Dimeloe S (2018). A transcriptionally and functionally distinct PD-1(+) CD8(+) T cell pool with predictive potential in non-small-cell lung cancer treated with PD-1 blockade. Nat Med.

[10] Grossman JE, Vasudevan D, Joyce CE, Hildago M (2021). Is PD-L1 a consistent biomarker for anti-PD-1 therapy? The model of balstilimab in a virally-driven tumor. Oncogene.

[11] Zou Y, Hu X, Zheng S, Yang A, Li X, Tang H (2021). Discordance of immunotherapy response predictive biomarkers between primary lesions and paired metastases in tumours: a systematic review and meta-analysis. EBioMedicine..

[12] McCarthy CE, White JM, Viola NT, Gibson HM (2020). In vivo imaging technologies to monitor the immune system. Front Immunol.

[13] Wahl RL, Jacene H, Kasamon Y, Lodge MA (2009). From RECIST to PERCIST: evolving considerations for PET response criteria in solid tumors. J Nucl Med.

[14] Wolchok JD, Hoos A, O'Day S, Weber JS, Hamid O, Lebbe C (2009). Guidelines for the evaluation of immune therapy activity in solid tumors: immune-related response criteria. Clin Cancer Res.

[15] Iafrate M, Fruhwirth GO (2020). How non-invasive in vivo cell tracking supports the development and translation of cancer immunotherapies. Front Physiol.

[16] Ashmore-Harris C, Iafrate M, Saleem A, Fruhwirth GO (2020). Non-invasive reporter gene imaging of cell therapies, including T cells and stem cells. Mol Ther.

[17] Wei W, Rosenkrans ZT, Liu J, Huang G, Luo QY, Cai W (2020). ImmunoPET: concept, design, and applications. Chem Rev.

[18] Larimer BM, Wehrenberg-Klee E, Caraballo A, Mahmood U (2016). Quantitative CD3 PET imaging predicts tumor growth response to anti-CTLA-4 therapy. J Nucl Med.

[19] Mayer KE, Mall S, Yusufi N, Gosmann D, Steiger K, Russelli L (2018). T-cell functionality testing is highly relevant to developing novel immuno-tracers monitoring T cells in the context of immunotherapies and revealed CD7 as an attractive target. Theranostics.

[20] Farwell MD, Gamache RF, Babazada H, Hellmann MD, Harding JJ, Korn R, et al. CD8-targeted PET imaging of tumor infiltrating T cells in patients with cancer: a phase I first-in-human study of (89)Zr-Df-IAB22M2C, a radiolabeled anti-CD8 minibody. J Nucl Med. 2021. 10.2967/jnumed.121.262485. 10.2967/jnumed.121.262485 PMC9051598 34413145

[21] Olafsen T, Wu AM (2010). Antibody vectors for imaging. Semin Nucl Med.

[22] Wu AM (2014). Engineered antibodies for molecular imaging of cancer. Methods.

[23] Tavare R, Escuin-Ordinas H, Mok S, McCracken MN, Zettlitz KA, Salazar FB (2016). An effective immuno-PET imaging method to monitor CD8-dependent responses to immunotherapy. Cancer Res.

[24] Seo JW, Tavare R, Mahakian LM, Silvestrini MT, Tam S, Ingham ES (2018). CD8(+) T-cell density imaging with (64)Cu-labeled Cys-diabody informs immunotherapy protocols. Clin Cancer Res.

[25] D'Huyvetter M, Xavier C, Caveliers V, Lahoutte T, Muyldermans S, Devoogdt N (2014). Radiolabeled nanobodies as theranostic tools in targeted radionuclide therapy of cancer. Expert Opin Drug Deliv.

[26] Fang J, Nakamura H, Maeda H (2011). The EPR effect: unique features of tumor blood vessels for drug delivery, factors involved, and limitations and augmentation of the effect. Adv Drug Deliv Rev.

[27] Salvador JP, Vilaplana L, Marco MP (2019). Nanobody: outstanding features for diagnostic and therapeutic applications. Anal Bioanal Chem.

[28] Rashidian M, Ingram JR, Dougan M, Dongre A, Whang KA, LeGall C (2017). Predicting the response to CTLA-4 blockade by longitudinal noninvasive monitoring of CD8 T cells. J Exp Med.

[29] Zhao H, Wang C, Yang Y, Sun Y, Wei W, Wang C (2021). ImmunoPET imaging of human CD8(+) T cells with novel (68)Ga-labeled nanobody companion diagnostic agents. J Nanobiotechnol.

[30] Feldwisch J, Tolmachev V, Lendel C, Herne N, Sjoberg A, Larsson B (2010). Design of an optimized scaffold for affibody molecules. J Mol Biol.

[31] Gonzalez Trotter DE, Meng X, McQuade P, Rubins D, Klimas M, Zeng Z (2017). In vivo imaging of the programmed death ligand 1 by (18)F PET. J Nucl Med.

[32] Sorensen J, Sandberg D, Sandstrom M, Wennborg A, Feldwisch J, Tolmachev V (2014). First-in-human molecular imaging of HER2 expression in breast cancer metastases using the 111In-ABY-025 affibody molecule. J Nucl Med.

[33] Fu R, Carroll L, Yahioglu G, Aboagye EO, Miller PW (2018). Antibody Fragment and affibody immunoPET imaging agents: radiolabelling strategies and applications. ChemMedChem.

[34] Tolmachev V, Orlova A (2020). Affibody molecules as targeting vectors for PET imaging. Cancers (Basel)..

[35] Krekorian M, Fruhwirth GO, Srinivas M, Figdor CG, Heskamp S, Witney TH (2019). Imaging of T-cells and their responses during anti-cancer immunotherapy. Theranostics.

[36] Sharpe AH, Pauken KE (2018). The diverse functions of the PD1 inhibitory pathway. Nat Rev Immunol.

[37] Heskamp S, Wierstra PJ, Molkenboer-Kuenen JDM, Sandker GW, Thordardottir S, Cany J (2019). PD-L1 microSPECT/CT imaging for longitudinal monitoring of PD-L1 expression in syngeneic and humanized mouse models for cancer. Cancer Immunol Res.

[38] Natarajan A, Mayer AT, Xu L, Reeves RE, Gano J, Gambhir SS (2015). Novel radiotracer for immunoPET imaging of PD-1 checkpoint expression on tumor infiltrating lymphocytes. Bioconjugate Chem.

[39] Niemeijer AN, Leung D, Huisman MC, Bahce I, Hoekstra OS, van Dongen G (2018). Whole body PD-1 and PD-L1 positron emission tomography in patients with non-small-cell lung cancer. Nat Commun.

[40] Syn NL, Teng MWL, Mok TSK, Soo RA (2017). De-novo and acquired resistance to immune checkpoint targeting. Lancet Oncol.

[41] Leach DR, Krummel MF, Allison JP (1996). Enhancement of antitumor immunity by CTLA-4 blockade. Science.

[42] Higashikawa K, Yagi K, Watanabe K, Kamino S, Ueda M, Hiromura M (2014). 64Cu-DOTA-anti-CTLA-4 mAb enabled PET visualization of CTLA-4 on the T-cell infiltrating tumor tissues. PLoS ONE..

[43] Andrews LP, Marciscano AE, Drake CG, Vignali DA (2017). LAG3 (CD223) as a cancer immunotherapy target. Immunol Rev.

[44] Lecocq Q, Awad RM, De Vlaeminck Y, De Mey W, Ertveldt T, Goyvaerts C (2021). Nanobody nuclear imaging allows noninvasive quantification of LAG-3 expression by tumor-infiltrating leukocytes and predicts response of immune checkpoint blockade. J Nucl Med..

[45] Triebel F, Jitsukawa S, Baixeras E, Roman-Roman S, Genevee C, Viegas-Pequignot E (1990). LAG-3, a novel lymphocyte activation gene closely related to CD4. J Exp Med.

[46] Huang CT, Workman CJ, Flies D, Pan X, Marson AL, Zhou G (2004). Role of LAG-3 in regulatory T cells. Immunity.

[47] Keane C, Law SC, Gould C, Birch S, Sabdia MB, Merida de Long L (2020). LAG3: a novel immune checkpoint expressed by multiple lymphocyte subsets in diffuse large B-cell lymphoma. Blood Adv..

[48] Gourd K (2017). ESMO 2017 Congress. Lancet Oncol.

[49] Lecocq Q, Zeven K, De Vlaeminck Y, Martens S, Massa S, Goyvaerts C (2019). Noninvasive imaging of the immune checkpoint LAG-3 using nanobodies, from development to pre-clinical use. Biomolecules..

[50] Dong D, Zheng L, Lin J, Zhang B, Zhu Y, Li N (2019). Structural basis of assembly of the human T cell receptor-CD3 complex. Nature.

[51] Ledbetter JA, Gentry LE, June CH, Rabinovitch PS, Purchio AF (1987). Stimulation of T cells through the CD3/T-cell receptor complex: role of cytoplasmic calcium, protein kinase C translocation, and phosphorylation of pp60c-src in the activation pathway. Mol Cell Biol.

[52] Beckford Vera DR, Smith CC, Bixby LM, Glatt DM, Dunn SS, Saito R (2018). Immuno-PET imaging of tumor-infiltrating lymphocytes using zirconium-89 radiolabeled anti-CD3 antibody in immune-competent mice bearing syngeneic tumors. PLoS ONE..

[53] Anasetti C, Martin PJ, Storb R, Appelbaum FR, Beatty PG, Davis J (1992). Treatment of acute graft-versus-host disease with a nonmitogenic anti-CD3 monoclonal antibody. Transplantation.

[54] Van Wauwe JP, De Mey JR, Goossens JG. Pillars article: OKT3: a monoclonal anti-human T lymphocyte antibody with potent mitogenic properties. J Immunol. 1980. 124:2708–13. J Immunol. 2016;197(9):3431–6. 27824589

[55] Stillwell R, Bierer BE (2001). T cell signal transduction and the role of CD7 in costimulation. Immunol Res.

[56] Selvaraj P, Plunkett ML, Dustin M, Sanders ME, Shaw S, Springer TA (1987). The T lymphocyte glycoprotein CD2 binds the cell surface ligand LFA-3. Nature.

[57] Timonen T, Gahmberg CG, Patarroyo M (1990). Participation of CD11a-c/CD18, CD2 and RGD-binding receptors in endogenous and interleukin-2-stimulated NK activity of CD3-negative large granular lymphocytes. Int J Cancer.

[58] Sasada T, Reinherz EL (2001). A critical role for CD2 in both thymic selection events and mature T cell function. J Immunol.

[59] Rooney MS, Shukla SA, Wu CJ, Getz G, Hacohen N (2015). Molecular and genetic properties of tumors associated with local immune cytolytic activity. Cell.

[60] Mall S, Yusufi N, Wagner R, Klar R, Bianchi H, Steiger K (2016). Immuno-PET imaging of engineered human T cells in tumors. Cancer Res.

[61] Griessinger CM, Maurer A, Kesenheimer C, Kehlbach R, Reischl G, Ehrlichmann W (2015). 64Cu antibody-targeting of the T-cell receptor and subsequent internalization enables in vivo tracking of lymphocytes by PET. Proc Natl Acad Sci USA.

[62] Cohen CJ, Zhao Y, Zheng Z, Rosenberg SA, Morgan RA (2006). Enhanced antitumor activity of murine-human hybrid T-cell receptor (TCR) in human lymphocytes is associated with improved pairing and TCR/CD3 stability. Cancer Res.

[63] Barat B, Kenanova VE, Olafsen T, Wu AM (2011). Evaluation of two internalizing carcinoembryonic antigen reporter genes for molecular imaging. Mol Imaging Biol.

[64] Larimer BM (2018). Reporter genes for PET imaging of CAR T cells offers insight into adoptive cell transfer. J Nucl Med.

[65] Kawai O, Ishii G, Kubota K, Murata Y, Naito Y, Mizuno T (2008). Predominant infiltration of macrophages and CD8(+) T cells in cancer nests is a significant predictor of survival in stage IV nonsmall cell lung cancer. Cancer.

[66] Galon J, Costes A, Sanchez-Cabo F, Kirilovsky A, Mlecnik B, Lagorce-Pages C (2006). Type, density, and location of immune cells within human colorectal tumors predict clinical outcome. Science.

[67] Zhang L, Conejo-Garcia JR, Katsaros D, Gimotty PA, Massobrio M, Regnani G (2003). Intratumoral T cells, recurrence, and survival in epithelial ovarian cancer. N Engl J Med.

[68] Clemente CG, Mihm MC, Bufalino R, Zurrida S, Collini P, Cascinelli N (1996). Prognostic value of tumor infiltrating lymphocytes in the vertical growth phase of primary cutaneous melanoma. Cancer.

[69] Tumeh PC, Harview CL, Yearley JH, Shintaku IP, Taylor EJ, Robert L (2014). PD-1 blockade induces responses by inhibiting adaptive immune resistance. Nature.

[70] Chen DS, Mellman I (2017). Elements of cancer immunity and the cancer-immune set point. Nature.

[71] Rosenberg JE, Hoffman-Censits J, Powles T, van der Heijden MS, Balar AV, Necchi A (2016). Atezolizumab in patients with locally advanced and metastatic urothelial carcinoma who have progressed following treatment with platinum-based chemotherapy: a single-arm, multicentre, phase 2 trial. Lancet.

[72] Ahmadzadeh M, Pasetto A, Jia L, Deniger DC, Stevanovic S, Robbins PF (2019). Tumor-infiltrating human CD4(+) regulatory T cells display a distinct TCR repertoire and exhibit tumor and neoantigen reactivity. Sci Immunol..

[73] Kagamu H, Kitano S, Yamaguchi O, Yoshimura K, Horimoto K, Kitazawa M (2020). CD4(+) T-cell Immunity in the peripheral blood correlates with response to anti-PD-1 therapy. Cancer Immunol Res.

[74] Yang Y, Kohler ME, Chien CD, Sauter CT, Jacoby E, Yan C (2017). TCR engagement negatively affects CD8 but not CD4 CAR T cell expansion and leukemic clearance. Sci Transl Med..

[75] Pandit-Taskar N, Postow MA, Hellmann MD, Harding JJ, Barker CA, O'Donoghue JA (2020). First-in-humans imaging with (89)Zr-Df-IAB22M2C anti-CD8 minibody in patients with solid malignancies: preliminary pharmacokinetics, biodistribution, and lesion targeting. J Nucl Med.

[76] Lohrmann C, O'Reilly EM, O'Donoghue JA, Pandit-Taskar N, Carrasquillo JA, Lyashchenko SK (2019). Retooling a blood-based biomarker: phase I assessment of the high-affinity CA19-9 antibody HuMab-5B1 for immuno-PET imaging of pancreatic cancer. Clin Cancer Res.

[77] Grimfors G, Schnell PO, Holm G, Johansson B, Mellstedt H, Pihlstedt P (1989). Tumour imaging of indium-111 oxine-labelled autologous lymphocytes as a staging method in Hodgkin's disease. Eur J Haematol.

[78] Fisher B, Packard BS, Read EJ, Carrasquillo JA, Carter CS, Topalian SL (1989). Tumor localization of adoptively transferred indium-111 labeled tumor infiltrating lymphocytes in patients with metastatic melanoma. J Clin Oncol.

[79] Auletta S, Iodice V, Galli F, Lepareur N, Devillers A, Signore A (2018). Study of binding kinetics and specificity of (99m)Tc-SSS-complex and (99m)Tc-HMPAO to blood cells. Contrast Media Mol Imaging.

[80] Biancone L, Schillaci O, Capoccetti F, Bozzi RM, Fina D, Petruzziello C (2005). Technetium-99m-HMPAO labeled leukocyte single photon emission computerized tomography (SPECT) for assessing Crohn's disease extent and intestinal infiltration. Am J Gastroenterol.

[81] Man F, Lim L, Volpe A, Gabizon A, Shmeeda H, Draper B (2019). In vivo PET tracking of (89)Zr-labeled Vgamma9Vdelta2 T cells to mouse xenograft breast tumors activated with liposomal alendronate. Mol Ther.

[82] Braunlein E, Lupoli G, Fuchsl F, Abualrous ET, de Andrade Kratzig N, Gosmann D (2021). Functional analysis of peripheral and intratumoral neoantigen-specific TCRs identified in a patient with melanoma. J Immunother Cancer..

[83] Signore A, Annovazzi A, Barone R, Bonanno E, D'Alessandria C, Chianelli M (2004). 99mTc-interleukin-2 scintigraphy as a potential tool for evaluating tumor-infiltrating lymphocytes in melanoma lesions: a validation study. J Nucl Med.

[84] D'Alessandria C, di Gialleonardo V, Chianelli M, Mather SJ, de Vries EF, Scopinaro F (2010). Synthesis and optimization of the labeling procedure of 99mTc-HYNIC-interleukin-2 for in vivo imaging of activated T lymphocytes. Mol Imaging Biol.

[85] Markovic SN, Galli F, Suman VJ, Nevala WK, Paulsen AM, Hung JC (2018). Non-invasive visualization of tumor infiltrating lymphocytes in patients with metastatic melanoma undergoing immune checkpoint inhibitor therapy: a pilot study. Oncotarget.

[86] Hartimath SV, Draghiciu O, van de Wall S, Manuelli V, Dierckx RA, Nijman HW (2017). Noninvasive monitoring of cancer therapy induced activated T cells using [(18)F]FB-IL-2 PET imaging. Oncoimmunology..

[87] van de Donk PP, Wind TT, Hooiveld-Noeken JS, van der Veen EL, Glaudemans A, Diepstra A (2021). Interleukin-2 PET imaging in patients with metastatic melanoma before and during immune checkpoint inhibitor therapy. Eur J Nucl Med Mol Imaging..

[88] Mulder WM, Bloemena E, Stukart MJ, Kummer JA, Wagstaff J, Scheper RJ (1997). T cell receptor-zeta and granzyme B expression in mononuclear cell infiltrates in normal colon mucosa and colon carcinoma. Gut.

[89] Mahrus S, Craik CS (2005). Selective chemical functional probes of granzymes A and B reveal granzyme B is a major effector of natural killer cell-mediated lysis of target cells. Chem Biol.

[90] Larimer BM, Wehrenberg-Klee E, Dubois F, Mehta A, Kalomeris T, Flaherty K (2017). Granzyme B PET imaging as a predictive biomarker of immunotherapy response. Cancer Res.

[91] Larimer BM, Bloch E, Nesti S, Austin EE, Wehrenberg-Klee E, Boland G (2019). The effectiveness of checkpoint inhibitor combinations and administration timing can be measured by granzyme B PET imaging. Clin Cancer Res.

[92] Boros E, Holland JP (2018). Chemical aspects of metal ion chelation in the synthesis and application antibody-based radiotracers. J Label Compd Radiopharm.

[93] Price EW, Orvig C (2014). Matching chelators to radiometals for radiopharmaceuticals. Chem Soc Rev.

[94] Mu L, Hohne A, Schubiger PA, Ametamey SM, Graham K, Cyr JE (2008). Silicon-based building blocks for one-step 18F-radiolabeling of peptides for PET imaging. Angew Chem Int Ed Engl.

[95] Becaud J, Mu L, Karramkam M, Schubiger PA, Ametamey SM, Graham K (2009). Direct one-step 18F-labeling of peptides via nucleophilic aromatic substitution. Bioconjugate Chem.

[96] McBride WJ, Sharkey RM, Goldenberg DM (2013). Radiofluorination using aluminum-fluoride (Al18F). EJNMMI Res.

[97] Russelli L, Martinelli J, De Rose F, Reder S, Herz M, Schwaiger M (2020). Room temperature Al(18) F labeling of 2-aminomethylpiperidine-based chelators for PET imaging. ChemMedChem.

[98] Cleeren F, Lecina J, Ahamed M, Raes G, Devoogdt N, Caveliers V (2017). Al(18)F-labeling of heat-sensitive biomolecules for positron emission tomography imaging. Theranostics.

[99] van der Veen EL, Suurs FV, Cleeren F, Bormans G, Elsinga PH, Hospers GAP (2020). Development and evaluation of interleukin-2-derived radiotracers for PET imaging of T cells in mice. J Nucl Med.

[100] Vugts DJ, Klaver C, Sewing C, Poot AJ, Adamzek K, Huegli S (2017). Comparison of the octadentate bifunctional chelator DFO*-pPhe-NCS and the clinically used hexadentate bifunctional chelator DFO-pPhe-NCS for (89)Zr-immuno-PET. Eur J Nucl Med Mol Imaging.

[101] Chomet M, Schreurs M, Bolijn MJ, Verlaan M, Beaino W, Brown K (2021). Head-to-head comparison of DFO* and DFO chelators: selection of the best candidate for clinical (89)Zr-immuno-PET. Eur J Nucl Med Mol Imaging.

[102] Kelly MP, Tavare R, Giurleo JT, Makonnen S, Hickey C, Danton MA, Arnold TC, Ma D, Dai J, Pei J, Kirshner JR, Olson WC, Thurston G. Immuno-PET detection of LAG-3 expressing intratumoral lymphocytes using the zirconium-89 radiolabeled fully human anti-LAG-3 antibody REGN3767. Cancer Res. 2018; Proceedings of the American Association for Cancer Research Annual Meeting 2018.

[103] Freise AC, Zettlitz KA, Salazar FB, Lu X, Tavare R, Wu AM (2017). ImmunoPET imaging of murine CD4(+) T cells using anti-CD4 Cys-diabody: effects of protein dose on T cell function and imaging. Mol Imaging Biol.

[104] Yusufi N, Mall S, Bianchi HO, Steiger K, Reder S, Klar R (2017). In-depth characterization of a TCR-specific tracer for sensitive detection of tumor-directed transgenic T CELLS BY IMMUno-PET. Theranostics.

[105] Rahmim A, Zaidi H (2008). PET versus SPECT: strengths, limitations and challenges. Nucl Med Commun.

[106] von Eyben FE, Baumann GS, Baum RP (2018). PSMA diagnostics and treatments of prostate cancer become mature. Clin Transl Imaging.

[107] Loubaki L, Tremblay T, Bazin R (2013). In vivo depletion of leukocytes and platelets following injection of T cell-specific antibodies into mice. J Immunol Methods.

[108] Cobbold SP, Jayasuriya A, Nash A, Prospero TD, Waldmann H (1984). Therapy with monoclonal antibodies by elimination of T-cell subsets in vivo. Nature.

[109] Wang Z, Han W (2018). Biomarkers of cytokine release syndrome and neurotoxicity related to CAR-T cell therapy. Biomark Res.

[110] Bagley J, Tian C, Sachs DH, Iacomini J (2002). T cells mediate resistance to genetically modified bone marrow in lethally irradiated recipients. Transplantation.

[111] Belka C, Ottinger H, Kreuzfelder E, Weinmann M, Lindemann M, Lepple-Wienhues A (1999). Impact of localized radiotherapy on blood immune cells counts and function in humans. Radiother Oncol.

[112] Yao Z, Jones J, Kohrt H, Strober S (2011). Selective resistance of CD44hi T cells to p53-dependent cell death results in persistence of immunologic memory after total body irradiation. J Immunol.

[113] Kachikwu EL, Iwamoto KS, Liao YP, DeMarco JJ, Agazaryan N, Economou JS (2011). Radiation enhances regulatory T cell representation. Int J Radiat Oncol Biol Phys.

[114] McBride WH, Chiang CS, Olson JL, Wang CC, Hong JH, Pajonk F (2004). A sense of danger from radiation. Radiat Res.

